# Direct photon production and PDF fits reloaded

**DOI:** 10.1140/epjc/s10052-018-5944-4

**Published:** 2018-06-09

**Authors:** John M. Campbell, Juan Rojo, Emma Slade, Ciaran Williams

**Affiliations:** 1 Fermilab, P.O. Box 500, Batavia, IL 60510 USA; 2 Department of Physics and Astronomy, VU University, 1081 HV Amsterdam, The Netherlands; 3 Nikhef Theory Group, Science Park 105, 1098 XG Amsterdam, The Netherlands; 4 Rudolf Peierls Centre for Theoretical Physics, University of Oxford, 1 Keble Road, Oxford, OX1 3NP UK; 5 Department of Physics, University at Buffalo, The State University of New York, Buffalo, 14260 USA

## Abstract

Direct photon production in hadronic collisions provides a handle on the gluon PDF by means of the QCD Compton scattering process. In this work we revisit the impact of direct photon production on a global PDF analysis, motivated by the recent availability of the next-to-next-to-leading (NNLO) calculation for this process. We demonstrate that the inclusion of NNLO QCD and leading-logarithmic electroweak corrections leads to a good quantitative agreement with the ATLAS measurements at 8 and 13 TeV, except for the most forward rapidity region in the former case. By including the ATLAS 8 TeV direct photon production data in the NNPDF3.1 NNLO global analysis, we assess its impact on the medium-*x* gluon. We also study the constraining power of the direct photon production measurements on PDF fits based on different datasets, in particular on the NNPDF3.1 no-LHC and collider-only fits. We also present updated NNLO theoretical predictions for direct photon production at 13 TeV that include the constraints from the 8 TeV measurements.

## Introduction

The determination of parton distribution functions (PDFs) of the proton [1–4] is an important component of the LHC program for many analyses, from precision tests of the Standard Model to searches for new physics beyond it. Within the global fitting framework, the gluon PDF has been traditionally constrained by the scaling violations of deep-inelastic scattering (DIS) structure functions and from inclusive jet production [5]. More recently, a number of additional collider observables have demonstrated their constraining power on the gluon PDF, from differential distributions in top-quark pair production [6] to the *Z* boson transverse momentum [7] and *D* meson production in the forward region [8, 9]. In this respect, the recent NNPDF3.1 global analysis [10] demonstrated how a robust determination of the medium and large-*x* NNLO gluon PDF can be achieved by the combination of LHC measurements of top-quark pair, *Z* $p_T$, and inclusive jet production – see also the discussion in [11].

**Fig. 1:**
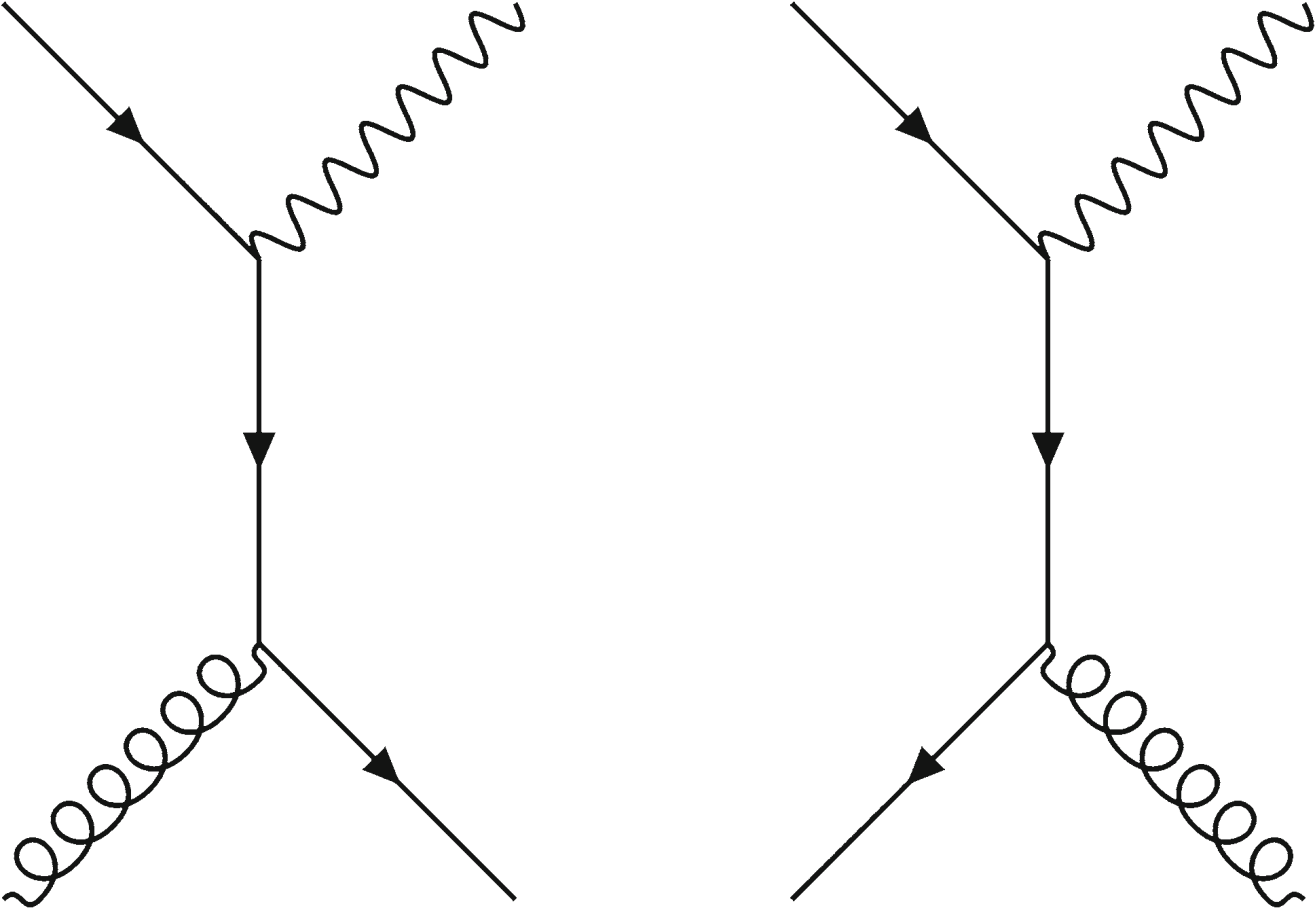
Feynman diagrams for direct photon production at leading order via the QCD Compton scattering process (left) and $q \bar{q}$ annihilation (right)

Another process that has been advocated to constrain the gluon in a global PDF analysis is direct (or “prompt”) photon production at hadron colliders. Indeed, direct photon production, $pp \rightarrow \gamma + X$, probes the gluon directly at leading order through the QCD Compton scattering process $qg \rightarrow \gamma q$ shown in Fig. 1. Taking into account the kinematics of available LHC data, direct photon measurements provide information on the gluon in the range between $x\simeq 10^{-3}$ and $x\simeq 0.1$ [12, 13]. In addition, direct photons can also be produced via quark–antiquark annihilation (also shown in Fig. 1), such that this process also allows us to probe the contribution of different quark flavours in the same *x* region.

However, exploiting collider measurements of direct photon production to constrain the gluon PDF is complicated by the fact that high-$E_T$ photons can also be produced via the collinear splitting of a final-state quark. These emissions have associated collinear singularities that are absorbed into non-perturbative quark-to-photon and gluon-to-photon fragmentation functions (FFs). This fragmentation component is only loosely constrained by LEP data [14], therefore inducing a potentially large source of theoretical uncertainty.

In spite of these complications, direct photon production data from fixed-target experiments were used in early global PDF fits, such as those of [15–17]. However, the increased availability of jet production data from the Tevatron, together with the difficulties in reconciling NLO QCD theory with some fixed-target measurements, led to the abandonment of using photon data to constrain the large-*x* gluon. However, this general feeling that direct photon production data was not suitable for PDF fits was demonstrated to be incorrect by the analysis of Ref. [13]. There it was shown that a good agreement between NLO theory and direct (isolated) photon production measurements could be obtained for a wide range of collider energies from RHIC and SPS to the Tevatron and the LHC at $\sqrt{s}=7$ TeV. This analysis also found that the LHC measurements lead to a moderate reduction of the gluon PDF uncertainties in the region around $x\simeq 0.02$.

Despite the results of this study, none of the most recently-updated global PDF fits [10, 18–20] include collider direct photon measurements. The reason for this is twofold. On the one hand, the NLO QCD calculations are affected by large scale uncertainties, thus making direct photon production inappropriate for NNLO global analyses that require precision theoretical predictions. On the other hand, in order to relate theory calculations with experimental measurements one needs to account for the poorly-understood fragmentation component.

The first of these objections was removed by the availability of the NNLO QCD calculation [21], which together with the corresponding electroweak corrections [22] was found to provide a good quantitative description of the ATLAS measurements at $\sqrt{s}=8$ TeV [23] at central photon rapidities [21]. The second objection can be somewhat alleviated by applying the smooth cone isolation prescription proposed by Frixione [24]. This isolation condition removes the need for fragmentation functions from the theoretical calculation, at the cost of introducing a difference between the isolation definitions used in the theoretical and the experimental analyses. However, as will be discussed later, this difference has been studied at NLO in great detail and found to be of limited practical consequence.

In addition to their relevance for PDF fits, photon production at the LHC is of great interest in searches for new physics beyond the standard model (BSM). For instance, recent searches for BSM physics with photons in the final state from ATLAS and CMS include searches for new particles by looking for high-mass resonances [25–27], anomalous couplings [28–31], and by measuring missing $E_T$ distributions [32–35]. These searches rely on a good understanding of the QCD background for photon production. It is therefore necessary to account for higher order QCD and electroweak corrections and to use recent global PDF fits that can properly model the background and signal events.

With this motivation, the goal of this paper is to revisit the impact of available LHC direct photon production measurements on a global NNLO PDF analysis. Specifically, we will include the ATLAS 8 TeV measurements [23] into the NNPDF3.1 analysis, in order to quantify the agreement between data and NNLO QCD theory and the corresponding impact on the gluon PDF. We find that a good description of this dataset is achieved, except for the most forward rapidity bin, and show that the inclusion of the photon data leads to a moderate reduction of the gluon PDF uncertainties at medium *x*. These fit results are cross-checked with those of the Monte Carlo Bayesian reweighting procedure [36, 37]. In addition, we aim to study the constraining power of the direct photon production measurements on PDF fits based on different datasets, in particular on the NNPDF3.1 no-LHC and collider-only sets. We also show that using state-of-the-art theory and including the constraints from the 8 TeV direct photon measurements leads to an excellent description of the recent ATLAS 13 TeV data [38].

The paper is organised as follows. In Sect. 2 we review existing measurements of direct photon production, focusing on the ATLAS data used in the present study. In Sect. 3 we discuss the theoretical setup for computing the theoretical predictions for direct photon production. The impact of the photon data upon the gluon PDF is presented in Sect. 4, and in Sect. 5 we provide updated predictions for direct photon production at 13 TeV. Finally, in Sect. 6 we conclude with a summary of the results and outline possible future developments. We assess the impact of the correlations among systematic uncertainties in Appendix A, and compare the results of the fits with those obtained with the Bayesian reweighting method in Appendix B.

## Experimental data

There exist many measurements of direct photon production both at fixed-target and at collider experiments (we refer the reader to ref. [13] for a detailed list). The measurements performed at the highest centre-of-mass energies are those from the LHC and from the Tevatron. At the Tevatron, the CDF and DØ  experiments have measured direct photon cross-sections at $\sqrt{s} = 630$ GeV [39, 40], 1.8 TeV [41–45] and 1.96 TeV [46–48]. More recently, the ATLAS and CMS experiments during Run I have performed similar measurements at 7 TeV [49–53] and ATLAS at 8 TeV [23], while thus far during Run II only ATLAS has measured direct photon production at 13 TeV [38].

In this work, we will concentrate on the ATLAS measurements at 8 and 13 TeV. The 8 TeV data exhibits reduced statistical and systematic uncertainties as compared to their 7 TeV counterparts [51, 53], and is thus suitable for inclusion in a global PDF analysis. The 13 TeV measurements will be used only to compare with our theoretical predictions, but will not be included in the fit since the experimental uncertainties are larger than the 8 TeV data due to the limited integrated luminosity, $\mathcal {L}_\mathrm{int}=3.2$ fb$^{-1}$.

The ATLAS 8 TeV direct photon production measurement is presented as differential distributions in the photon transverse energy ($E_T ^\gamma$) in four photon pseudorapidity ($\eta ^\gamma$) bins:

2.1 $$\begin{aligned} \begin{aligned}&\text {region 1:}\,\, 0< |\eta ^\gamma |< 0.6 , \\&\text {region 2:}\,\, 0.6 \le |\eta ^\gamma |< 1.37 , \\&\text {region 3:}\,\, 1.56 \le |\eta ^\gamma |< 1.81, \\&\text {region 4:}\,\, 1.81 \le |\eta ^\gamma | < 2.37 . \end{aligned}\end{aligned}$$

The measurements cover the transverse energy range $25< E_T ^\gamma < 1500$ GeV, though the upper limit is reduced in the more forward bins. As we will discuss in Sect. 3, the kinematic cuts applied constrain the number of points included in the fit to $N_\mathrm{dat}=49$.

For each of the experimental bins, the information on the statistical, total systematic, and luminosity uncertainties is provided by ATLAS. The full breakdown of the experimental systematic uncertainties including the information on cross-correlations corresponding to this measurement was only posted in HepData after the completion of the main results of this work. For this reason, the fits presented here are based on a $\chi ^2$ constructed by adding the total systematic and statistical uncertainties in quadrature. The luminosity uncertainty on the other hand is taken to be fully correlated among all the bins, and correlated to other ATLAS measurements at 8 TeV included in the PDF fit. In Appendix A we assess the impact that including the correlation between the experimental systematic uncertainties in the $\chi ^2$ definition has at the level of both PDFs and at the level of fit quality.

As will be discussed in Sect. 4, the most forward rapidity bin, $1.81 \le |\eta ^\gamma | < 2.37$, is excluded from the fit due to the tensions between the experimental data and the theoretical predictions. In this respect, the fact that the covariance matrix was not available at the time of the completion of the main results of this work implies that we cannot quantitatively study the origin of the tension in this forward bin. We discuss in Appendix A the description of the 4th rapidity bin upon the inclusion of the covariance matrix. Therefore, we have taken a conservative approach and excluded the anomalous bin; in Sect. 4.1 we discuss the impact in the fit of this bin and motivate in more detail its exclusion from our analysis.

**Fig. 2:**
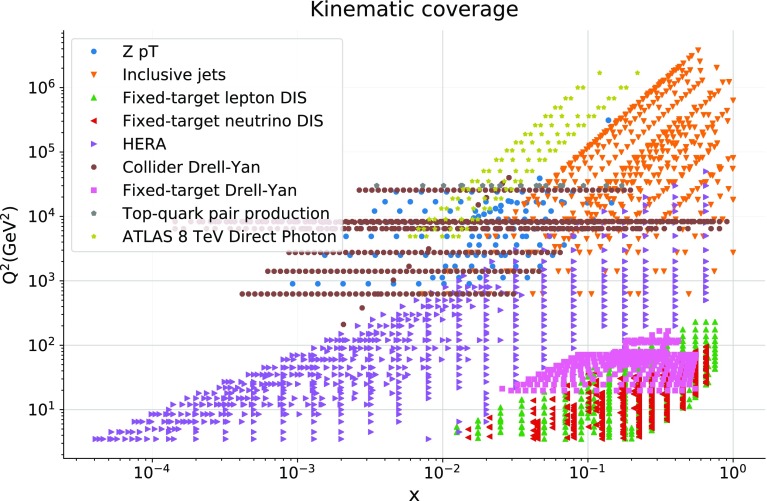
The coverage in the $(x, Q^2)$ kinematic plane of the 8 TeV ATLAS photon measurements (using LO kinematics), compared to the dataset used in the global NNPDF3.1 fit

In Fig. 2 we show the kinematic coverage of the ATLAS 8 TeV data in the $(x,Q^2)$ plane computed using LO kinematics, alongside with that of the dataset used in the global NNPDF3.1 fit. At LO one may write $x_\pm = 2 E_T^\gamma \, \exp {(\pm \eta ^\gamma )} / s$, so that each datapoint of the 8 TeV measurement corresponds to two points in the $(x,Q^2)$ plane. From this comparison, we observe that the photon data probes a $(x,Q^2)$ region only partly covered by other experiments; specifically the medium-*x* range for over two orders of magnitude in $Q^2$. Therefore including the photon data allows one to constrain a new kinematic region beyond the range of previous PDF fits.

Concerning the ATLAS 13 TeV measurements, the data is presented in the same format as at 8 TeV and covers a $E_T ^\gamma$ range between $125< E_T ^\gamma < 1500$ GeV, for a total of $N_\mathrm{dat}=53$ datapoints. The covariance matrix is constructed in the same way as for the 8 TeV data, namely by adding statistical and total systematic uncertainties in quadrature, and treating the luminosity uncertainty as fully correlated among all the bins.

In order to distinguish prompt photons (produced in the hard-scattering process) from secondary photons (which occur copiously in decays of hadrons) the experimental analyses apply isolation criteria to the measured photons. Since secondary photons are predominantly associated with a large amount of hadronic activity, the experiments restrict the hadronic radiation that is present in a cone around the photon candidate. The isolation requirement used in the ATLAS analysis is $E_T^\gamma$-dependent, optimised to obtain the best signal-to-background ratio. The additional advantage of the relatively tight isolation applied by ATLAS is that it significantly reduces the contribution from prompt photons which are produced in the fragmentation of a hard parton. These contributions also have significant hadronic activity near the photon and are hence suppressed by the isolation condition.

## Theoretical setup

In this section we outline the NNLO QCD and LL electroweak calculations that are used to compare with the ATLAS direct photon production data. We also describe the settings adopted in the calculation of the NLO QCD predictions, in particular the fast NLO APPLgrid interpolation [54] that is required to include the photon data in the global analysis.

As discussed in the introduction, direct photon production in hadronic collisions can proceed via two different types of processes. The photon can either be directly emitted as part of the hard-scattering interaction, as in Fig. 1, or alternatively can be produced via the collinear fragmentation of a parton. Taking into account these two contributions, the differential cross-section as a function of $E_T ^\gamma$ can be written as

3.1 $$\begin{aligned} \mathrm{d}\sigma&= \mathrm{d}\sigma _\text {dir} + \mathrm{d}\sigma _\text {frag}\nonumber \\&= \sum _{a,b = q, \bar{q}, g} \int \mathrm{d}x_a \mathrm{d}x_b f_a(x_a; \mu _F^2) f_b(x_b;\mu _F^2)\nonumber \\&\quad \times \Bigg [\mathrm{d}\hat{\sigma } ^\gamma _{ab} (p_\gamma , x_a, x_b; \mu _R, \mu _F, \mu _\text {ff}) \nonumber \\&+ \sum _{c=q,\bar{q} ,g} \int _{z_\text {min}} ^1 \frac{\mathrm{d}z}{z^2} \mathrm{d}\hat{\sigma }^c _{ab} (p_\gamma ,x_a,x_b,z;\mu _R, \mu _F, \mu _\text {ff})\nonumber \\&\quad \times D_c ^\gamma ( z; \mu _\text {ff}^2)\Bigg ], \end{aligned}$$

where $D_c ^\gamma (z,\mu _\text {ff}^2)$ is the fragmentation function of a parton *c* to a photon carrying momentum fraction *z* and $f_a(x_a; \mu _F^2)$ is the PDF of a parton *a*. While $\mu _R$ and $\mu _F$ are the standard renormalization and factorization scales, note the appearance of a new scale $\mu _\text {ff}$, known as the fragmentation scale. The NNLO QCD corrections to the direct component of the partonic cross-section $\hat{\sigma } ^\gamma _{ab}$ have been computed in [21], while the fragmentation component $\hat{\sigma }^c _{ab}$ is only known at NLO.

The need to account for the fragmentation functions $D^\gamma _c$ can be eliminated by adopting the smooth cone isolation criterion [24],

3.2 $$\begin{aligned} \sum E_T^\text {had} (R)< \epsilon _\gamma E_T ^\gamma \left( \frac{1- \cos R}{1 - \cos R_0} \right) ^n \forall R < R_0 , \end{aligned}$$

with $E_T^\mathrm{had}$ being the hadronic transverse energy contained in a cone of radius *R* around a photon of $E_T^\gamma$, and where *n*, $R_0$ and $\epsilon _\gamma$ are parameters of the algorithm. Here the $(1- \cos R)$ term suppresses the collinear singularity present as $R\rightarrow 0$, but arbitrarily soft radiation is allowed inside the cone $R_0$ in order to preserve the cancellation of infrared poles in the calculation.

The granularity of an experimental calorimeter is such that this smooth cone isolation can never be directly replicated in experimental analyses, thereby introducing an unwelcome disconnect between theoretical calculations and the data. However, the parameters appearing in the above isolation definition, $\epsilon _\gamma$ and *n*, are arbitrary and this allows them to be tuned to replicate the features of a full calculation including fragmentation, Eq. (3.1). Such a study was first performed at NLO in [55], finding that the values $\epsilon _\gamma = 0.025$ and $n=2$ result in good agreement between the full calculation and the smooth cone result to within a few percent. A similar study was undertaken in the context of di-photon production at NNLO in [56], for which the parameters $n=2$ and $\epsilon _\gamma = 0.1$ were found to agree well with the fragmentation calculation.

The differences between the two types of isolation criteria were further studied recently at NLO in [57], finding a small ($\sim 2\%)$ correction that is independent of $E_T^\gamma$ over a range of values similar to the one studied in this paper. This correction is the same approximate size as the missing higher-order uncertainty associated to the NNLO calculation. A full quantitative description of these theoretical uncertainties is beyond the scope of this work, so we therefore do not account for the uncertainty due to the choice of isolation algorithm. In this work, we adopt the smooth cone isolation, Eq. (3.2), with parameters $n=2, \epsilon _\gamma = 0.1$ and $R_0 = 0.4$, and motivated by the above studies we assume that the residual uncertainties due to the choice of isolation prescription are negligible and should not have a bearing on the PDF fits that we perform.

In order to account for the impact of Sudakov effects induced by virtual loops of heavy electroweak gauge bosons, we include the resummation of the electroweak Sudakov logarithms at leading-logarithmic (LL) accuracy. Following the procedure in [22, 58], we set the QED coupling constant to be $\alpha _\mathrm{em}(m_Z) = 1/127.9$ in the calculation.

The electroweak effects may be accounted for by an overall rescaling of the cross-section of the form

3.3 $$\begin{aligned} \sigma ^{\left( \text {NNLO QCD + LL EW}\right) } = [1 + \Delta _V ^\mathrm{ew}(E_T^\gamma ,s)] \times \sigma ^{\left( \text {NNLO QCD}\right) } , \end{aligned}$$

where the LL electroweak correction $\Delta _V ^\mathrm{ew}(E_T^\gamma ,s)$ is given by [22, 58]

3.4 $$\begin{aligned}&\Delta _V ^\mathrm{ew}(E_T^\gamma ,s)\nonumber \\&\quad = \frac{1.72 -21.68 E_T^\gamma +12.16 (E_T^\gamma )^2 -3.05 (E_T^\gamma )^3}{1 - 2.3355 \cdot 10^{-2} y + 1.2310 \cdot 10^{-3} y^2},\nonumber \\ \end{aligned}$$

with $y \equiv (\sqrt{s}-7)/7$, and $\sqrt{s}$ being the hadronic center-of-mass energy expressed in TeV.

Since Eq. (3.4) is only valid for $E_T ^\gamma \gtrsim M_Z,M_W$, we include in the fit only data such that $E_T ^\gamma > 65$ GeV. This way, we can consistently use NNLO QCD and LL EW theory for all the data bins in the fit. In the central rapidity bin, this extra cut has the additional advantage of minimising the contribution from fragmentation photons, which are not included in our analysis, as the size of the fragmentation component decreases with $E_T^\gamma$. After applying this kinematic cut, we have $N_\mathrm{dat}=63$ datapoints to include in the fit. In addition, after removing the data from the most forward rapidity bin due to the poor $\chi ^2$, as mentioned in Sect. 2, we are left with $N_\mathrm{dat}=49$ datapoints.

Concerning the calculation of the NNLO QCD cross-sections, we start by computing theoretical predictions at NLO accuracy using MCFM [59] interfaced with LHAPDF6[60] and APPLgrid using the NNPDF3.1 NNLO PDF set [10] with the dynamical renormalization $(\mu _R)$ and factorization $(\mu _F)$ scales set equal to $E_T ^\gamma$. The output of this calculation is a fast NLO interpolation grid, as required for the inclusion of these measurements in a PDF fit. Subsequently, the NNLO QCD corrections from Ref. [21] are included in the form of bin-by-bin *K*-factors, defined as:

3.5 $$\begin{aligned} K \equiv \frac{\mathrm{d}\sigma ^\text {NNLO}}{\mathrm{d}E_T ^\gamma \mathrm{d}\eta ^\gamma }(\mathrm{NNLO~PDFs}) \bigg / \frac{\mathrm{d}\sigma ^\text {NLO}}{\mathrm{d}E_T ^\gamma \mathrm{d}\eta ^\gamma }(\mathrm{NNLO~PDFs}) ,\nonumber \\ \end{aligned}$$

so that only the perturbative order of the partonic cross-section is varied, but the PDFs are kept the same.

The technical details regarding this NNLO QCD computation can be found in the original publications [21, 57], and we refer the interested reader to these works for more detail. The only non-trivial change from these works is the manner in which the slicing variable $\tau _1^\mathrm{{cut}}$ is defined. In the original calculations this is set at a fixed value for the entire phase space, $\tau _1^\mathrm{{cut}}=0.08$ GeV. In the present analysis we instead use a dynamic cut that is determined by the photon transverse momentum, $\tau _1^\mathrm{{cut}} = 0.001 \times E_T^{\gamma }$. We find that this improves the overall performance of the computation, particularly in the determination of the NNLO corrections at high $E_T^{\gamma }$.

**Fig. 3:**
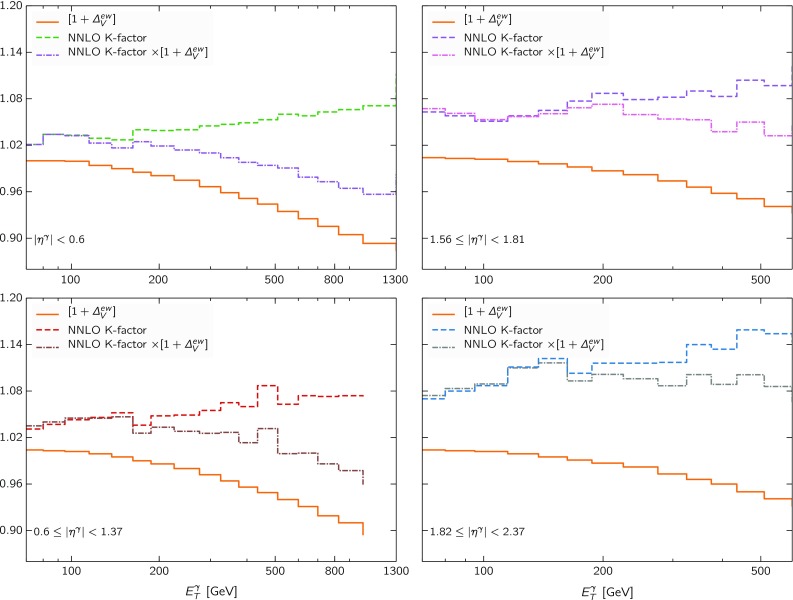
The NNLO QCD *K*-factor, Eq. (3.5), and the LL electroweak correction $[1 + \Delta _V ^\mathrm{ew}(E_T^\gamma ,s)]$, Eq. (3.4), in the four rapidity bins of the ATLAS 8 TeV measurement. We also show the results of its multiplicative combination, which indicates the overall correction applied to the NLO QCD cross-section

We show in Fig. 3 the NNLO QCD *K*-factors, Eq. (3.5), as well as the LL electroweak correction $[1 + \Delta _V ^\mathrm{ew}(E_T^\gamma ,s)]$, Eq. (3.4), in the four rapidity bins of the ATLAS 8 TeV measurement [23]. Both corrections become more important in the high $E_T^\gamma$ regions, where they deviate significantly from 1. We also show in Fig. 3 the results of the multiplicative combination of the NNLO QCD *K*-factor and of the LL EW effects, which represents the overall correction applied to the NLO QCD cross-section. We can observe that there is a partial cancellation between the two higher-order effects, since each pulls the NLO cross-section in an opposite direction.

As mentioned above, a fast interpolation of the NLO QCD calculation, required for the subsequent PDF fit, is constructed by interfacing APPLgrid with MCFM. These fast grids may be used to compute the cross-sections for any PDF set other than the one used in the original calculation with a very small calculational overhead. Specifically, we have used MCFM v6.8 interfaced with the MCFM/APPLgrid bridge code and with the HOPPET [61] PDF evolution program. Note that in this respect MCFM v6.8 had to be patched to reproduce the results of v8.0, which in turn correspond to the results of Ref. [21], as we verified explicitly.

**Fig. 4:**
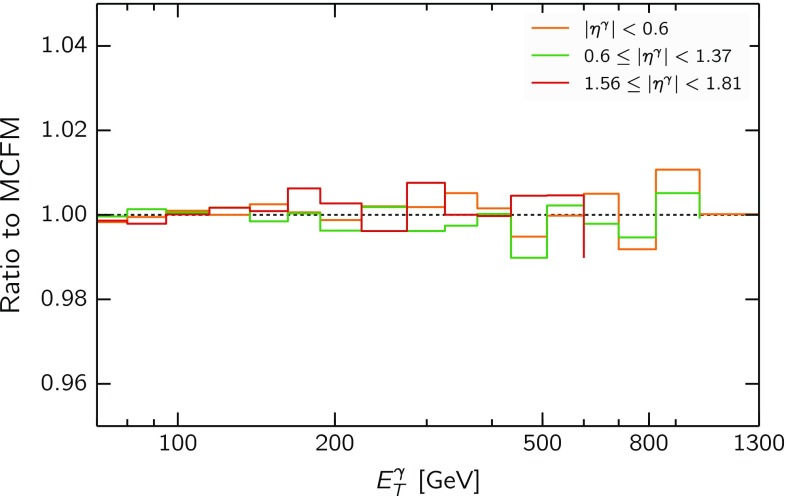
The ratio of the APPLgrid computations of the NLO QCD cross-section to the corresponding MCFM v6.8 result for the kinematics of the first three rapidity bins of the ATLAS 8 TeV measurement, using in both cases the NNPDF3.1 set as input

In order to obtain sufficiently high numerical precision, we ran MCFM in 10 batches with different random seeds and combined the resulting grids using the applgrid-combine script. This MCFM/APPLgrid computation was successfully benchmarked with the NLO code of Ref. [21], finding excellent agreement. In Fig. 4 we plot the ratio of the APPLgrid computations of the NLO QCD cross-section to the corresponding MCFM v6.8 result for the kinematics of the first three rapidity bins of the ATLAS 8 TeV measurement, using in both cases the NNPDF3.1 set as input. We find good agreement between the two methods within the uncertainties from the finite MC integration statistics, which are typically at the permille level.^1^

## Results

In this section we present the main results of this work, namely the impact of the ATLAS direct photon production data at $\sqrt{s}=8$ TeV on the NNPDF3.1 global analysis. NNPDF3.1 is the most up-to-date NNPDF release, including a wealth of new Tevatron and LHC datasets from processes such as Drell–Yan and $t\bar{t}$ pair-production and the transverse momentum of *Z* bosons. In contrast to previous fits, NNPDF3.1 independently parameterizes the charm content of the proton, eliminating any possible bias related to the assumption that the charm PDF is generated perturbatively [62].

In this context, an important difference of the present work as compared to the study of Ref. [13] is that the latter was based on the NNPDF2.1 fit, where the information on the gluon PDF was limited. This is not the case in NNPDF3.1, where the gluon PDF is already reasonably well constrained at medium and small-*x* from the combination of jet, $t\bar{t}$, and *Z* $p_T$ data, and therefore we expect the impact of the direct photon data on the gluon to be moderate.

Here we will also study the impact of the direct photon production data on fits based on reduced datasets, in particular the NNPDF3.1 no-LHC data and collider-only fits. We also compare other global PDF sets to the direct photon measurements, specifically MMHT14, CT14, and ABMP16. The corresponding comparisons with the ATLAS 13 TeV measurements as well as with the 13/8 cross-section ratio will then be presented in the next section.

### Comparison to the experimental data

**Fig. 5:**
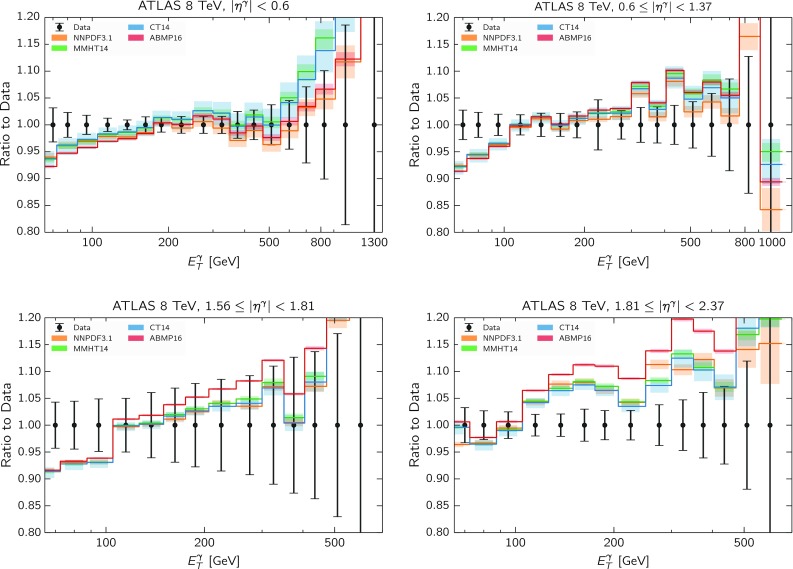
Comparison between the theoretical predictions for direct photon production data computed with different PDF sets and the ATLAS 8 TeV data, normalized to the central value of the former. The experimental statistical and systematic uncertainties have been added in quadrature. The error bands for the theory predictions include only the PDF uncertainties

To begin with, using the NNLO QCD theory supplemented with LL electroweak corrections described in Sect. 3, we have computed the differential cross-sections for the $E_T^{\gamma }$ distributions of the ATLAS 8 TeV measurement for different PDF sets. In all cases we use their default value for the strong coupling constant; for NNPDF3.1, MMHT14 and CT14 this is $\alpha _s(m_Z) = 0.118$ and for ABMP16, $\alpha _s(m_Z) = 0.1147$. In Fig. 5 we show the comparison of these theoretical predictions normalized to the central value of the ATLAS measurements, where the error bars on the experimental data are the sum in quadrature of the statistical and systematic uncertainties, while the error bands for the theory predictions include only the PDF uncertainties.

From the comparisons of Fig. 5 we see that across the first three rapidity bins, the various sets are in good agreement; in particular in the 3rd bin, NNPDF, CT14 and MMHT14 are very close to each other. We also find that the NNPDF3.1 and ABMP16 sets lead to a better description of the high $E_T^\gamma$ region in the central bin. On the other hand, one can clearly observe in the most forward bin a large disagreement between theory and data, in particular for ABMP16.

These trends are further examined in Table 1, where we compare the total $\chi ^2 /N_\text {dat}$ for the different PDF sets. We note that in this $\chi ^2$ computation the *experimental* definition of the covariance matrix is used [63], as opposed to the $t_0$ definition [64] which is only used during the fitting. From Table 1 we see that none of the four PDF sets manage to describe the most forward rapidity bin in a satisfactory way. We have verified that this is still the case even when this bin is included in the PDF fit. We show in Appendix A that even upon the inclusion of the covariance matrix, the poor description of the $\chi ^2$ still exists in the 4th rapidity bin. We therefore exclude this 4th rapidity bin from the analysis.

Then in Table 2 we show the same $\chi ^2$ comparison but now using only NLO QCD theory and without the LL electroweak corrections. In this case we find that the $\chi ^2$ in all bins is rather poor. Interestingly, the most forward rapidity bin exhibits a slight improvement in the $\chi ^2$ values, which however, remain large. As the most forward rapidity bin corresponds to the small-*x* region of one of the incoming partons, it would be interesting to verify if the theoretical description of this bin would be improved by including NLL*x* resummation of direct photon production, similar as what was done in [65, 66] for the HERA data. For the three rapidity bins used in the fit, one finds a dramatic improvement in the description of the data upon including the higher-order QCD and EW effects. This comparison highlights the phenomenological importance of the recent NNLO QCD calculation, and why only now we can robustly include the direct photon measurements into the global PDF fits.

**Table 1:** The $\chi ^2 /N_\text {dat}$ values of the 8 TeV ATLAS using NNLO QCD theory supplemented with LL electroweak corrections for different PDF sets. We provide the results for the four individual rapidity bins in Eq. (2.1), as well as their sum with and without the most forward bin

	$$\chi ^2 /N_\text {dat}$$					
	1st bin	2nd bin	3rd bin	4th bin	Total	Total excluding 4th bin
NNPDF3.1	0.81	1.61	0.89	1.97	1.83	1.12
MMHT14	1.94	2.49	1.02	2.19	2.31	1.89
CT14	1.63	2.18	0.96	2.02	2.01	1.65
ABMP16	1.38	2.70	1.27	7.78	3.50	1.91

**Table 2:** Same as in Table 1 but with only NLO QCD theory and without LL electroweak corrections

	$$\chi ^2 /N_\text {dat}$$					
	1st bin	2nd bin	3rd bin	4th bin	Total	Total excluding 4th bin
NNPDF3.1	1.55	2.35	1.44	1.83	1.69	1.71
MMHT14	3.37	3.43	1.57	2.08	2.73	2.87
CT14	2.91	3.03	1.51	1.99	2.51	2.57
ABMP16	2.48	4.19	2.03	3.19	2.59	2.53

### Impact on the global fit

In the following, we denote by NNPDF3.1 + ATLAS$\gamma$ the results of the fit obtained by adding the ATLAS 8 TeV direct photon production cross-sections to the NNPDF3.1 NNLO global analysis. In Table 3 we compare the resulting values of $\chi ^2/N_\mathrm{dat}$ for each of the three rapidity bins included in the fit as well as for their total. We find that the inclusion of the ATLAS direct photon data improves the agreement between the theoretical predictions and the experimental measurements, with the total $\chi ^2 /N_\text {dat}$ decreasing from 1.12 down to 0.96. This improvement is particularly marked in the second rapidity bin, where $\chi ^2 /N_\text {dat}$ is reduced from 1.61 to 1.37.

These results suggest that the ATLAS photon measurements seem to be consistent with the rest of the datasets in NNPDF3.1. In order to further investigate this issue, and to determine if the ATLAS photon measurements are in tension with some of the other datasets included in the fit, In Table 4 we provide the breakdown of the $\chi ^2/N_\mathrm{dat}$ values for the individual datasets, comparing the results from the NNPDF3.1 and NNPDF3.1 + ATLAS$\gamma$ sets.

We observe that the overall fit quality upon inclusion of the photon data is unchanged within statistical fluctuations. In addition, we find that the direct photon data does not appear to exhibit any tensions with existing datasets. In particular, there are no tensions with other datasets which constrain the gluon, such as top-quark pair and inclusive jets production and the *Z* transverse momentum distributions. This stability is further highlighted by the comparison in Table 5, where we have grouped datasets together in families of related processes. We find that the largest improvements in the values of the $\chi ^2/N_\mathrm{dat}$ indeed correspond to those processes with sensitivity to the gluon PDF. We can thus conclude that the constraints on the gluon from direct photon production are consistent with those of the rest of the datasets in NNPDF3.1.

**Fig. 6:**
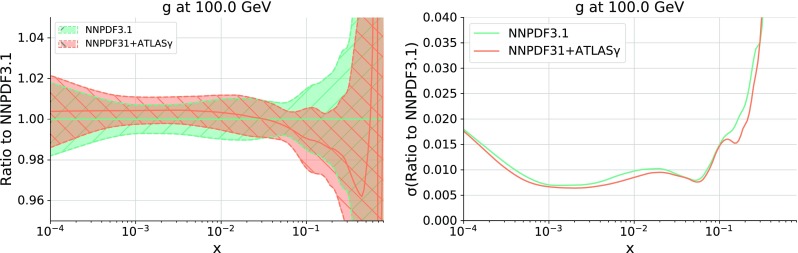
Left: comparison of the gluon PDF at $Q = 100$ GeV between the NNPDF3.1 and NNPDF3.1 + ATLAS$\gamma$ fits, normalized to the central value of the former. Right: the corresponding relative one-sigma PDF uncertainties in each case

In order to quantify the impact of the ATLAS direct photon data into the PDFs, in Fig. 6 we show the comparison of the gluon PDF at $Q = 100$ GeV between the NNPDF3.1 and NNPDF3.1 + ATLAS$\gamma$ fits, normalized to the central value of the former. In the same figure, we also compare the corresponding relative one-sigma PDF uncertainties in both cases. We find two main implications of adding the photon data into NNPDF3.1. The first one is a moderate reduction of the gluon PDF uncertainties in the region , which is consistent with the kinematic coverage spanned by the ATLAS measurements shown in Fig. 2.

The second is a downward shift of the gluon central value in the large-*x* region, by an amount of up to two thirds of the PDF uncertainty. For instance at $x\simeq 0.4$ the gluon in NNPDF3.1 + ATLAS$\gamma$ is about 4% smaller than in NNPDF3.1. Interestingly, the same trend was observed when adding top-quark pair differential distributions to NNPDF3.0 [6]. The overall consistency of the ATLAS direct photon data with the NNPDF3.1 dataset is highlighted by the fact that in the whole range of *x* the two fits are consistent within uncertainties.

**Fig. 7:**
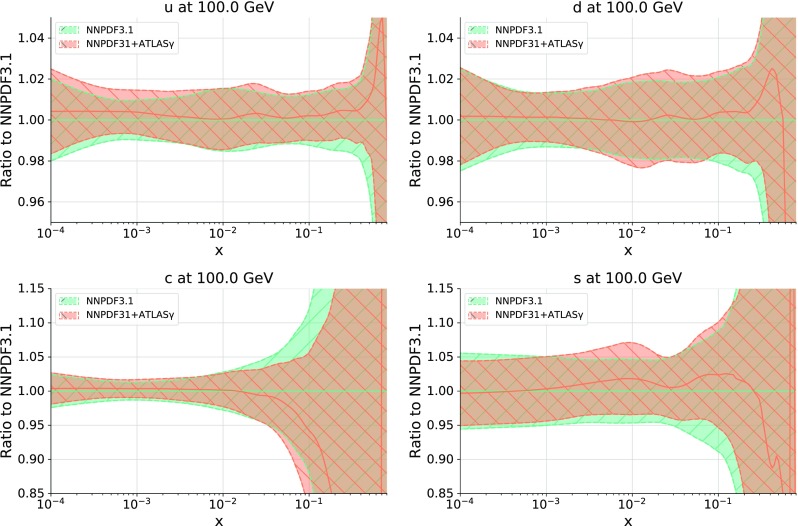
Comparison of the quark PDFs at $Q = 100$ GeV between the NNPDF3.1 and NNPDF3.1 + ATLAS$\gamma$ fits, normalized to the central value of the former

In addition to the impact of the photon data on the gluon, it is important to determine if the new data is consistent with the quark PDFs. In Fig. 7 we show the comparison of the quark PDFs at $Q = 100$ GeV between the NNPDF3.1 and NNPDF3.1 + ATLAS$\gamma$ fits. We find only rather small changes upon the addition of the photon data, both in terms of central values and of uncertainties, The exception is the charm PDF, which decreases in uncertainty across the full *x* range, partly due to its relation to the gluon via perturbative evolution. We therefore conclude that the ATLAS data does not introduce tensions with the quark PDFs, and furthermore does not strongly impact the size of their respective uncertainties.

**Table 3:** The $\chi ^2 /N_\text {dat}$ values for the 8 TeV ATLAS data for the NNPDF3.1 and NNPDF3.1 + ATLAS$\gamma$ fits, both for three rapidity bins included in the fit and for their total

	$$\chi ^2 /N_\text {dat}$$			
	1st bin	2nd bin	3rd bin	Total
NNPDF3.1	0.81	1.61	0.89	1.12
NNPDF31+ATLAS$\gamma$	0.66	1.37	0.82	0.96

**Table 4:** The values of $\chi ^2/N_\mathrm{dat}$ for all the datasets included in the present analysis, comparing the results from the NNPDF3.1 and NNPDF3.1 + ATLAS$\gamma$ fits. The corresponding values for the ATLAS 8 TeV direct photon data are reported in Table 3

Dataset	NNPDF3.1	NNPDF3.1 + ATLAS$\gamma$
NMC	1.30	1.28
SLAC	0.75	0.75
BCDMS	1.21	1.22
CHORUS	1.11	1.11
NuTeV dimuon	0.82	0.81
HERA I + II inclusive	1.16	1.16
HERA $\sigma _c^\mathrm{NC}$	1.45	1.45
HERA $F_2^b$	1.11	1.10
DY E866 $\sigma ^d_\mathrm{DY}/\sigma ^p_\mathrm{DY}$	0.41	0.46
DY E886 $\sigma ^p$	1.43	1.41
DY E605 $\sigma ^p$	1.21	1.21
CDF *Z* rap	1.48	1.49
CDF Run II $k_t$ jets	0.87	0.86
D0 *Z* rap	0.60	0.60
D0 $W\rightarrow e\nu$ asy	2.70	2.73
D0 $W\rightarrow \mu \nu$ asy	1.56	1.56
ATLAS total	1.09	1.07
ATLAS *W*, *Z* 7 TeV 2010	0.96	0.96
ATLAS high-mass DY 7 TeV	1.54	1.61
ATLAS low-mass DY 2011	0.90	0.91
ATLAS *W*, *Z* 7 TeV 2011	2.14	2.05
ATLAS jets 2010 7 TeV	0.94	0.92
ATLAS jets 2.76 TeV	1.03	1.01
ATLAS jets 2011 7 TeV	1.07	1.07
ATLAS *Z* $p_T$ 8 TeV $(p_T^{ll},M_{ll})$	0.93	0.93
ATLAS *Z* $p_T$ 8 TeV $(p_T^{ll},y_{ll})$	0.94	0.88
ATLAS $\sigma _{tt}^\mathrm{tot}$	0.86	1.09
ATLAS $t\bar{t}$ rap	1.45	1.39
CMS total	1.06	1.04
CMS *W* asy 840 pb	0.78	0.78
CMS *W* asy 4.7 fb	1.75	1.76
CMS Drell–Yan 2D 2011	1.27	1.29
CMS *W* rap 8 TeV	1.01	1.06
CMS jets 7 TeV 2011	0.84	0.82
CMS jets 2.76 TeV	1.03	1.00
CMS *Z* $p_T$ 8 TeV $(p_T^{ll},M_{ll})$	1.32	1.33
CMS $\sigma _{tt}^\mathrm{tot}$	0.20	0.24
CMS $t\bar{t}$ rap	0.94	0.93
LHCb total	1.47	1.42
LHCb *Z* 940 pb	1.49	1.49
LHCb $Z\rightarrow ee$ 2 fb	1.14	1.16
LHCb $W,Z \rightarrow \mu$ 7 TeV	1.76	1.69
LHCb $W,Z \rightarrow \mu$ 8 TeV	1.37	1.30
Total dataset	1.148	1.146

**Table 5:** Same as Table 4 now with individual experiments grouped into families of processes

	NNPDF3.1	NNPDF3.1 + ATLAS$\gamma$
Fixed-target lepton DIS	1.207	1.203
Fixed-target neutrino DIS	1.081	1.087
HERA	1.166	1.169
Fixed-target Drell–Yan	1.241	1.242
Collider Drell–Yan	1.356	1.346
Top-quark pair production	1.065	1.049
Inclusive jets	0.939	0.915
*Z* $p_T$	0.997	0.980
Total dataset	1.148	1.146

**Fig. 8:**
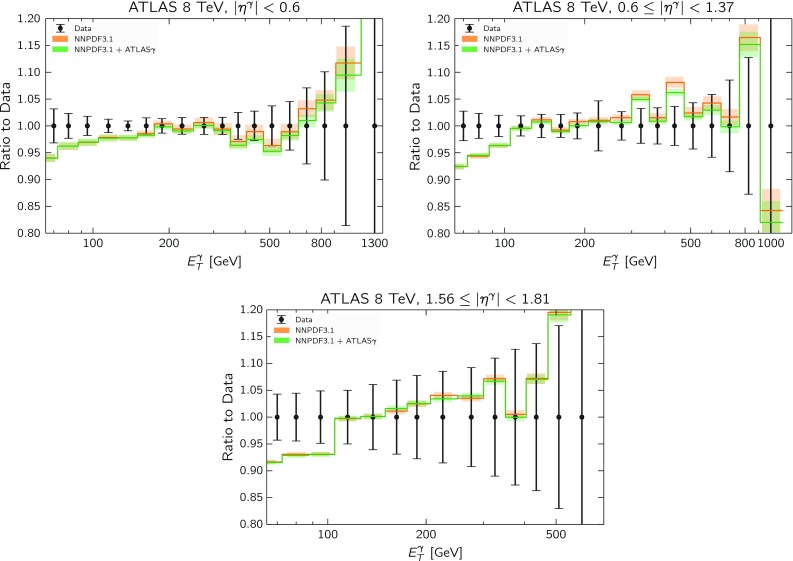
Same as Fig. 5, now comparing the NNPDF3.1 and NNPDF3.1 + ATLAS$\gamma$ sets for the three rapidity bins of the ATLAS 8 TeV data included in the fit

Finally, in Fig. 8 we show the same comparison between theory predictions and experimental data as in Fig. 5 now for the NNPDF3.1 and NNPDF3.1 + ATLAS$\gamma$ sets for the three rapidity bins of the ATLAS 8 TeV data included in the fit. We can see how in this case the predictions obtained with NNPDF3.1 + ATLAS$\gamma$ as an input move closer to the central values of the experimental data as compared to the NNPDF3.1 baseline, although by a small amount. These findings are consistent with the corresponding variations at the PDF level discussed in Figs. 6 and 7.

### Impact on fits based on reduced datasets

In addition to assessing the impact of the ATLAS direct photon production data when added to the NNPDF3.1 dataset, we have also studied its impact on fits using reduced datasets, specifically the NNPDF3.1 collider-only fit and no-LHC fits. The former excludes all DIS and Drell–Yan fixed-target data, with the motivation that collider observables might be cleaner and under better theoretical control, while the latter excludes all LHC measurements for specific applications such as in searches for BSM physics.

In the two cases, we find a good overall agreement between theory and data, as indicated in Table 6. For the fit without LHC data, the total $\chi ^2/N_\mathrm{dat}$ is reduced from 1.49 to 1.00. Recall that in this fit the constraints on the medium and large-*x* gluon are much looser, basically coming only from the Tevatron jet data, and thus one expects the impact of the ATLAS direct photon data to be more significant. For the collider only fit, the total $\chi ^2/N_\mathrm{dat}$ is already very good to begin with, 0.94, and is further reduced to $\chi ^2/N_\mathrm{dat}=0.87$ upon the addition of the photon data. This moderate improvement is consistent with the fact that the bulk of the gluon-sensitive datasets in NNPDF3.1 are already included in the collider-only dataset. Another interesting result from Table 6 is that in all cases an improved description of the three rapidity bins is obtained.

**Table 6:** Same as Table 3, now for the NNPDF3.1 fits based on reduced datasets

PDF set	$\chi ^2 /N_\text {dat}$			
	1st bin	2nd bin	3rd bin	Total
NNPDF3.1 no LHC data	1.26	2.07	0.96	1.49
NNPDF3.1 no LHC data + ATLAS$\gamma$	0.66	1.39	0.84	1.00
NNPDF3.1 collider only	0.89	1.29	0.68	0.94
NNPDF3.1 collider only + ATLAS$\gamma$	0.92	1.15	0.64	0.87

**Fig. 9:**
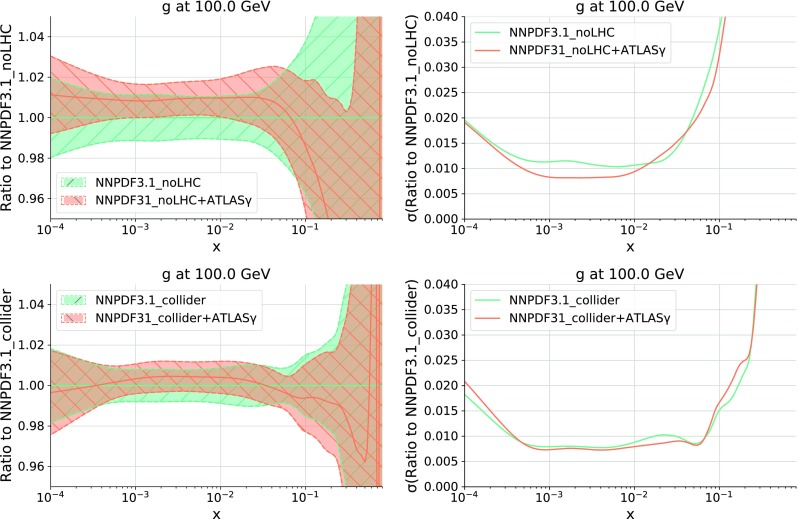
Same as Fig. 6 for the NNPDF3.1 no-LHC (upper) and collider-only (lower plots) fits

Next in Fig. 9 we show the same comparisons as in Fig. 6 but now for the NNPDF3.1 no-LHC and collider-only fits. In the case of the no-LHC fit, we find that the impact of adding the ATLAS photon data is larger than in the global fit, both in terms of the shift in the gluon central values and in the reduction of its PDF uncertainties. This is consistent with the fact that $g(x,Q^2)$ is less constrained in the no-LHC fit than in the global fit. The trend in central values is the same for the collider-only fits: moderate enhancement at medium *x* followed by a suppression at large *x*. The impact of the ATLAS photon data is also moderate at the level of PDF uncertainties in the collider-only fit.

To summarize, the results of this study demonstrate that the qualitative impact of the ATLAS 8 TeV direct photon production data on the gluon PDF in the fits based on reduced datasets is consistent with that of the global analysis. In all cases, we find an improvement in the quantitative description of the ATLAS data, as shown in Table 6. Interestingly, we also find that the direct photon data prefer a softer gluon at large *x* irrespective of the input dataset used, a trend that is similar to the one induced by the top-quark pair differential cross-sections.

**Table 7:** Same as Table 1 for the ATLAS 13 TeV direct photon production measurements

PDF set	$\chi ^2 /N_\text {dat}$				
	1st bin	2nd bin	3rd bin	4th bin	Total
NNPDF3.1	0.68	0.53	0.28	0.47	0.65
NNPDF3.1 + ATLAS$\gamma$	0.70	0.49	0.30	0.46	0.65
MMHT14	0.81	0.73	0.27	0.45	0.70
CT14	0.75	0.65	0.28	0.41	0.64
ABMP16	0.82	0.89	0.20	1.56	1.05

## Direct photon production at 13 TeV

In this section we present the comparison between state-of-the-art theoretical predictions and experimental data for the recent ATLAS measurements of direct photon production at 13 TeV [38]. The motivation is two-fold. On the one hand, we want to verify whether or not we can quantitatively describe direct photon production at 13 TeV, and in particular understand if the disagreement found for the most forward bin at 8 TeV (see Sect. 4.1) is also present at a higher center-of-mass energy. On the other hand, we aim to provide predictions for direct photon production at 13 TeV that include the constraints from the same process at 8 TeV: we will do this by using the NNPDF3.1 + ATLAS$\gamma$ fit constructed in the previous section.

To begin with, in Table 7 we provide the $\chi ^2/N_\mathrm{dat}$ values for different NNLO PDF sets to the ATLAS 13 TeV measurements using the theory settings described in Sect. 3. We also include here the predictions using the NNPDF3.1 + ATLAS$\gamma$ set, which accounts for the constraints of the 8 TeV photon measurements. We find that the different PDF sets provide an equally satisfactory description of this dataset, with the total $\chi ^2/N_\mathrm{dat}\simeq 1$ in all cases. In particular, we find an excellent description of the most forward rapidity bin (with the exception perhaps of ABMP16), in contrast to what was found at 8 TeV. One should note, however, that this measurement is based on a relatively small integrated luminosity, $\mathcal {L}_\mathrm{int}=3.2$ fb$^{-1}$, and therefore its uncertainties are larger than for the 8 TeV case, explaining the reduced discrimination power.

**Fig. 10:**
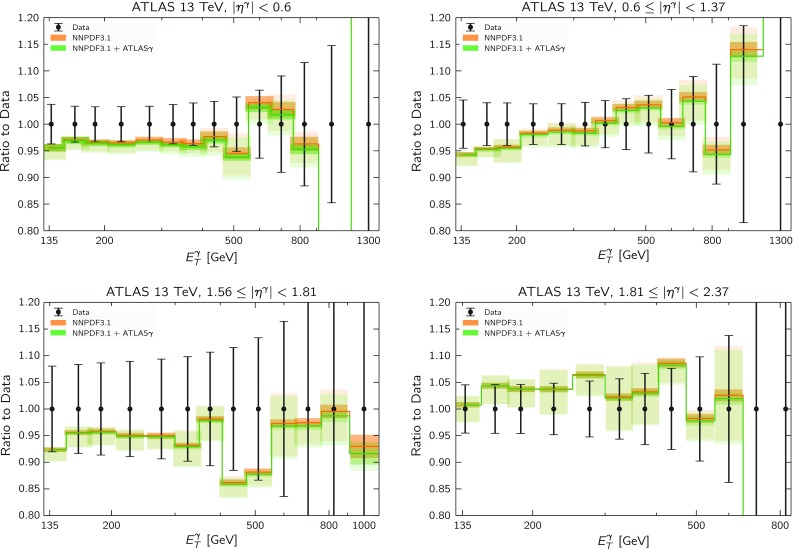
Same as Fig. 8 for the ATLAS 13 TeV direct photon measurements. In addition to the PDF uncertainties shown in the previous cases (darker bands), here we also include the scale uncertainties associated to the NNLO QCD calculation (lighter bands)

As can be seen from Table 7, the differences in the values of $\chi ^2$ between NNPDF3.1 and NNPDF3.1 + ATLAS$\gamma$ are small. This may be further observed in Fig. 10, where we compare the theory predictions for the 13 TeV data with both NNPDF3.1 and NNPDF3.1 + ATLAS$\gamma$. In addition to the PDF uncertainties shown in the previous cases (darker bands), here we also include the scale uncertainties associated with the NNLO QCD calculation (lighter bands), as discussed below. The two PDF sets are in good agreement with each other and the limited statistics of the measurement do not allow us to discriminate among them. This can also be seen from the fact that the experimental uncertainties are significantly larger than the differences between the two theoretical predictions. It is also interesting to take a closer look at the most forward rapidity bin of the 13 TeV measurement, which in the 8 TeV case had to be excluded from the fit. Here instead we find reasonably good agreement between theory and data, although again, there are larger experimental errors in this bin and therefore one cannot conclude that the description of the 13 TeV data is better than at 8 TeV.

As mentioned above, we also indicate in Fig. 10 the scale uncertainties associated with the NNLO QCD calculation (shown as the lighter error bands) in addition to the standard PDF uncertainties. These scale uncertainties have been estimated using the standard practice of independently varying the renormalization $\mu _R$ and factorization $\mu _F$ scales by a factor of two. For the majority of $E_T^\gamma$ bins, the scale uncertainty is $\mathcal {O}(5\%)$, reaching a maximum of $\mathcal {O}(10\%)$ in the most forward rapidity bin at high $E_T^\gamma$. At NLO, we find the typical size of the scale uncertainty to be approximately double that of the NNLO one, thus compounding the requirement to have the NNLO predictions in order to adequately describe the direct photon data.

One of the main differences that arises in the comparison between data and theory at 8 and 13 TeV, as we discussed, is that the most forward rapidity bin is poorly described in the former case, while it is reasonably well described in the latter. A possible way forward to understand the origin of this discrepancy is to take ratios of the cross-section measurements at the two centre-of-mass energies. Such ratios are useful since many theoretical and experimental systematic uncertainties cancel out [67], allowing us to elucidate possible issues arising for individual center-of-mass energies. With this motivation, we have constructed the following ratio

5.1 $$\begin{aligned} R_{13/8}(E_T^\gamma ,\eta ^\gamma ) \equiv \frac{ d\sigma (E_T^\gamma ,\eta ^\gamma ) }{ dE_T^\gamma d\eta ^\gamma }\Bigg |_{13~\mathrm{TeV}}\Bigg / \frac{ d\sigma (E_T^\gamma ,\eta ^\gamma ) }{ dE_T^\gamma d\eta ^\gamma }\Bigg |_{8~\mathrm{TeV}} , \end{aligned}$$

for those bins where both $E_T^\gamma$ and $\eta ^\gamma$ overlap between the two center of mass energies, corresponding to a total of 47 bins. Since the experimental covariance matrix is not available at 13 TeV and the description of the 4th rapidity bin is poor at 8 TeV both with and without the covariance matrix, the uncertainty on the ratio Eq. (5.1) is obtained by adding in quadrature the total experimental errors in the numerator and the denominator. For the theoretical calculation of Eq. (5.1), the correlation between the PDF uncertainties at 8 and 13 TeV is accounted for.

**Fig. 11:**
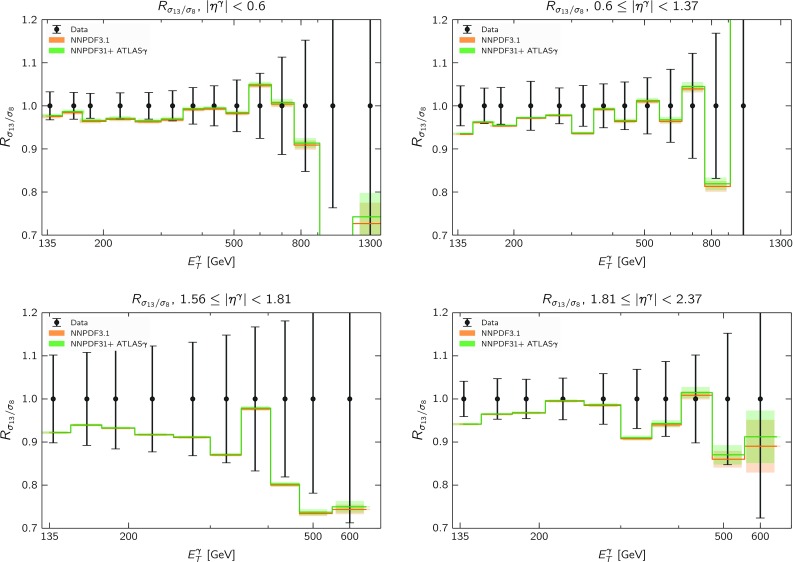
Comparison between the experimental measurements of the $R_{13/8}(E_T^\gamma ,\eta ^\gamma )$ ratio and the corresponding theoretical calculations using NNPDF3.1 and NNPDF3.1 + ATLAS$\gamma$, normalized to the central experimental value. The theory band includes only the contribution from the PDF uncertainties

In Fig. 11 we show a comparison between the experimental measurements of the $R_{13/8}(E_T^\gamma ,\eta ^\gamma )$ ratio, Eq. (5.1), with the corresponding calculations using the NNPDF3.1 and NNPDF3.1 + ATLAS$\gamma$ sets, normalized to the central value of the experimental data. Here the theoretical uncertainty band includes only the contribution from the PDF uncertainties. From this comparison we find that there is good agreement between data and theory for all the bins, including for the most forward rapidity bin which was problematic at 8 TeV. The results of Fig. 11 suggest that the underlying reason for the disagreement at 8 TeV in the most forward bin, either an inadequacy of the theory calculation or some issue with the experimental measurement, is a common effect between the two center of mass energies which mostly cancels out when computing their ratio.

**Fig. 12:**
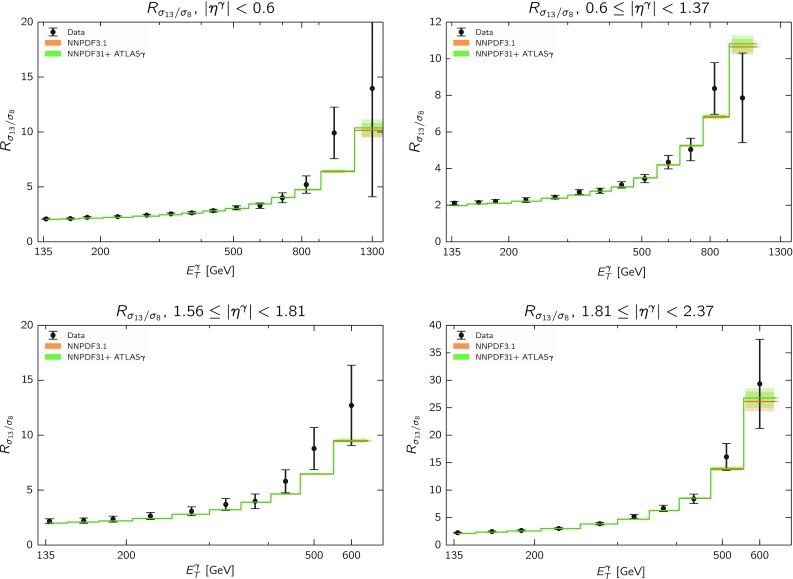
Same as in Fig. 11 without normalizing to the experimental data

In order to further understand how the cross-section ratio Eq. (5.1) behaves as a function of $E_T^\gamma$ and $\eta ^\gamma$, in Fig. 12 we show the same comparison as in Fig. 11 but this time without normalizing to the experimental data. We can see that there is excellent agreement between theory and data in all rapidity bins, both at low and high values of $E_T^\gamma$; moreover, we can also observe that the trend in the data-theory agreement is consistent across all the rapidity bins. These results therefore compound the argument that there is some inadequacy in either the theory or the experimental analysis for the most forward bin at $\sqrt{s}=8$ TeV.

In order to substantiate this point, in Table 8 we provide the $\chi ^2/N_\mathrm{dat}$ values for the ratio of cross-sections between 13 and 8 TeV, Eq. (5.1), with different input PDFs. As we can see from this comparison, all PDF sets are in agreement with the cross-section ratios, both for the total dataset and for the individual rapidity bins. In particular, we find that the description of the cross-section ratio $R_{13/8}$ in the most forward bin is the best with NNPDF3.1 + ATLAS$\gamma$. Table 8 provides further evidence that the origin of the disagreement between theory and data at 8 TeV in this bin is common with the 13 TeV case, since it mostly cancels in the ratio.

## Summary

The quantitative understanding of the detailed features of photon production at the LHC is of crucial importance for a wide range of analyses, from searches for Higgs decays and BSM resonances to precision Standard Model measurements. In this work, we have revisited the possibility of using direct photon production from the LHC to constrain the parton distribution functions of the proton within a global QCD fit. By using state-of-the-art NNLO QCD calculations combined with LL electroweak corrections, we have quantified the impact of the ATLAS 8 TeV photon production data on the gluon PDF from the NNPDF3.1 global analysis.

Our results indicate that the LHC direct photon production data leads to both a moderate reduction of the gluon uncertainties at medium-*x* and a preference for a somewhat softer central value at large-*x*. These effects are more marked when the direct photon data is added on top of fits based on reduced datasets, in particular the NNPDF3.1 no-LHC fit. We have also demonstrated that including both NNLO QCD and LL electroweak corrections is required in order to achieve a quantitative agreement with the experimental data for the entire kinematic range in $E_T^\gamma$ and $\eta ^\gamma$. Moreover, we find that the constraints from the direct photon data are consistent with those of other gluon-sensitive measurements included in NNPDF3.1 such as the *Z* $p_T$, inclusive jets, and $t\bar{t}$ differential distributions.

Here we have also provided theoretical predictions for the ATLAS measurements of direct photon production at 13 TeV as well as for the ratio of cross-sections between 13 and 8 TeV. In this case, we find that due to the relatively small integrated luminosity used for the 13 TeV measurement, its discrimination power is rather limited. It would therefore be important to repeat the 13 TeV analysis using the full integrated luminosity of Run II, in order to complement the information provided by the 8 TeV data. In this respect, it is essential that the experimental collaborations make public the covariance matrices of their measurements, else their lack of availability limits the physics output that can be extracted from their own data.

Our results demonstrate that there is no reason, neither in principle nor in practice, for excluding collider direct photon data from a global PDF analysis. Indeed, the most precise LHC measurements available agree well with state-of-the-art theoretical predictions, and the latter can be included in global PDF analyses using fast interpolation tables. The information provided by the ATLAS 8 TeV direct photon measurements turns out to be consistent with the constraints provided by other gluon-sensitive datasets included in NNPDF3.1, and leads to a moderate reduction of the gluon uncertainties. For these reasons, collider direct photon production should be rightfully restored to its well-deserved position as a full member of the global PDF analysis toolbox.

**Table 8:** Same as Table 1 for the ratio of cross-sections between 13 and 8 TeV, Eq. (5.1)

PDF set	$\chi ^2 /N_\text {dat}$				
	1st bin	2nd bin	3rd bin	4th bin	Total
NNPDF3.1	0.66	0.75	0.79	0.45	0.68
NNPDF3.1 + ATLAS$\gamma$	0.58	0.72	0.77	0.41	0.64
MMHT14	0.96	0.85	0.83	0.50	0.82
CT14	0.90	0.80	0.80	0.52	0.79
ABMP16	0.84	0.90	0.95	0.69	0.87

The main output of this work, the NNPDF3.1 + ATLAS$\gamma$ NNLO fit, is available in the LHAPDF6 format [60] from the NNPDF collaboration webpage

http://nnpdf.mi.infn.it/for-users/unpolarized-pdf-sets/

with the file name NNPDF31_nnlo_as_0118_directphoton. In addition, the fast NLO tables computed using MCFM and APPLgrid for the ATLAS 8 and 13 TeV direct photon measurements produced in this work, together with the corresponding NNLO/NLO *K*-factors Eq. (3.5), are also publicly available from the same website.

## Figures and Tables

**Fig. 13:**
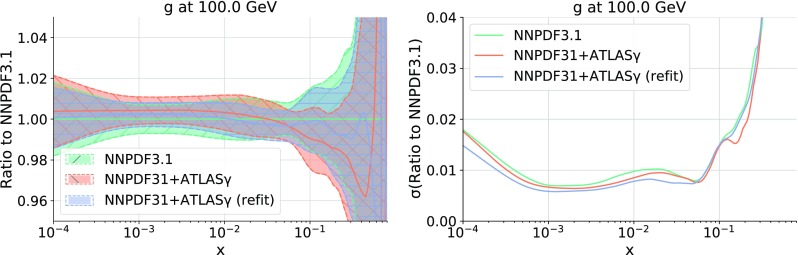
Same as Fig. 6, now adding to the comparison the results of the fit including the correlations between the experimental systematic uncertainties (labelled as “refit”)

**Fig. 14:**
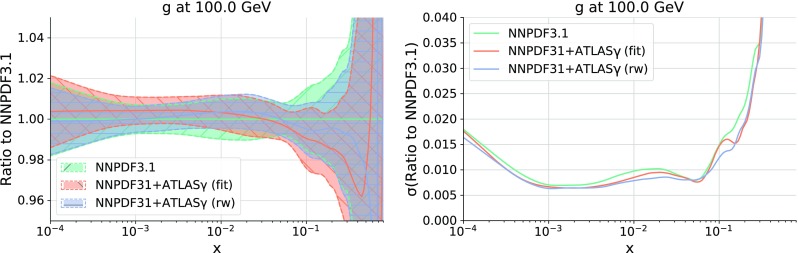
Same as Fig. 6 now adding to the comparison the results of the NNPDF3.1 + ATLAS$\gamma$(rw) set obtained by applying the Bayesian reweighting procedure

## A The impact of correlations in the systematic uncertainties

The baseline results of this work, presented in Sect. 4, have been obtained by adding in quadrature the statistical and experimental uncertainties, except for the luminosity uncertainty which is treated as fully correlated among all the data bins. As mentioned in Sect. 2, we only realised after the completion of this work that the full breakdown of the experimental systematic uncertainties corresponding to the ATLAS 8 TeV measurement had just been posted in HepData. The same breakdown of the experimental systematic uncertainties, however, is not yet available for the 13 TeV data. In this appendix, we study the impact of accounting for the effects of the correlations among the systematic uncertainties of the 8 TeV ATLAS data both at the level of parton distributions and of the values of the $\chi ^2$.

We will consider here two scenarios for the correlation model. In the first one, all sources of systematic uncertainty are fully correlated among the data bins. In the second one, a subset of sources of systematic errors will be considered as uncorrelated between data bins. Specifically, using the notation used in the corresponding HepData entry,^2^ the following sources are treated as point-to-point uncorrelated: sysPhotonID, sysPhotonIsolation, sysBackgroundID, sysBackgroundIsolation sysEnergyResolution. The reason for these two choices is that, initially, the ATLAS analyzers recommended to treat all errors as fully correlated, but at a later stage they provided us with an updated recommendation for their correlation model based on the decorrelation of some systematic sources.

Let us first of all discuss the impact of the experimental correlations at the PDF level. We will show results with the first correlation model, where all sources of systematic uncertainties are fully correlated bin-to-bin. In Fig. 13 we show the same comparison as in Fig. 6, now adding the results of the fit including the correlations between the experimental systematic uncertainties (labelled as “refit”). Reassuringly, the results are reasonably similar as compared to the fit where these correlations are neglected, though there are some small differences. In particular, concerning the PDF central values, the shift as compared to NNPDF3.1 is reduced once the correlations are accounted for. At the level of uncertainties, we also find a similar pattern as the one shown in Fig. 6, with the main differences being that the PDF error reduction is a bit more marked for  and is somewhat less important for larger values of *x*. All in all, the qualitative impact of the ATLAS 8 TeV direct photon data on the PDFs is similar irrespective of whether or not one includes the information on correlations in the $\chi ^2$ definition.

Turning now to the comparison at the fit quality level, in Table 9 we show the values of the $\chi ^2$ per data point including the information on the correlation of systematic uncertainties.

We display the results for three fits: the baseline NNPDF3.1, the NNPDF3.1 + ATLAS$\gamma$ fit presented in Sect. 4, and the corresponding fit using the correlated $\chi ^2$ for the minimisation (labelled as “refit”) and shown in Fig. 13. Note that the first two of these fits have been obtained from the minimisation of $\chi ^2$ which does not include the information on the correlations of experimental systematic uncertainties. The consistent values of the $\chi ^2/N_\mathrm{dat}$ for these two fits were reported in Table 3, where excellent agreement with the data was found.

**Table 9:** The values of the $\chi ^2$ per data point including the information on the correlation of systematic uncertainties. We show the results for three fits: the baseline NNPDF3.1, the NNPDF3.1 + ATLAS$\gamma$ fit presented in Sect. 4, and the corresponding fit using the correlated $\chi ^2$ for the minimisation (labelled as “refit”) and shown in Fig. 13. Note that the first two of these fits have been obtained from the minimisation of $\chi ^2$ which does not include the information on the correlations of systematic uncertainties

	$$\chi ^2 /N_\text {dat}$$			
	1st bin	2nd bin	3rd bin	Total
NNPDF3.1	1.52	3.95	2.57	3.03
NNPDF31+ATLAS$\gamma$	1.40	3.90	2.53	3.02
NNPDF31+ATLAS$\gamma$ (refit)	1.51	3.93	2.57	3.00

From the comparison between the results of Tables 9 and 3 we find that the fit quality is poorer once the information on correlated systematics is accounted for. In particular one gets a total $\chi ^2/N_\mathrm{dat}\simeq 3$ for the NNPDF3.1 + ATLAS$\gamma$ fit as compared to $\chi ^2/N_\mathrm{dat}\simeq 1$ in the corresponding case where these correlations are neglected. The description of the first rapidity bin is still satisfactory, but not that of the second and third rapidity bins. While the origin of these poor $\chi ^2$ values is still not understood, similar issues have been reported in the case of the ATLAS inclusive jet measurements by different groups (see e.g. [10, 68]). Here we have tried to vary the nominal correlation model, for instance by neglecting the correlations between different rapidity bins, but this does not modify the numbers in Table 9 in any significant way.

We have also verified that the most forward rapidity bin is still very poorly described once the full information on experimental correlations is taken into account. Using NNPDF3.1 as input, one finds $\chi ^2/N_\mathrm{dat}\simeq 5.2$ for the fourth rapidity bin of the ATLAS 8 TeV measurement, a number which is mostly unchanged when this bin is included in the fit. This result provides further evidence in support of our decision to exclude this bin from our baseline fits.

Let us now turn to discuss the case of the second correlation model mentioned above, namely the one where a selected number of sources of systematic error are treated as uncorrelated. In Table 10 we show the same comparison as in Table 9 where now the numbers in the second row have been computed using the partial decorrelation model for the experimental covariance matrix. We can observe a marked improvement as compared to the case where all systematic errors are treated as fully correlated among data bins, although the data/theory agreement is still not ideal. This result suggests that a further study of the correlation model of this measurement might further improve the numerical agreement between theory and data, for example in the case of a partial decorrelation of some of the other systematic sources. Such a study, which might be advantageous also for other LHC measurements such as jet production, is however beyond the scope of this paper.

**Table 10:** Same as Table 9, where now the numbers in the second row have been computed using the partial decorrelation model for the experimental covariance matrix. See text for more details

	$$\chi ^2 /N_\text {dat}$$			
	1st bin	2nd bin	3rd bin	Total
NNPDF3.1	1.52	3.95	2.57	3.03
NNPDF3.1 (part. decorr.)	1.09	2.64	1.88	1.98

**Table 11:** Same as Table 3, now with the $\chi ^2/N_\mathrm{dat}$ values corresponding to the reweighted NNPDF3.1 + ATLAS$\gamma$ set. We also provide the value of $N_\mathrm{eff}$ corresponding to the total dataset

	$\chi ^2 /N_\text {dat}$			$N_\text {eff}$
	NNPDF3.1	NNPDF3.1 + ATLAS$\gamma$ (fit)	NNPDF3.1 + ATLAS$\gamma$ (rw)	
1st bin	0.81	0.66	0.71	–
2nd bin	1.61	1.37	1.53	–
3rd bin	0.89	0.82	0.88	–
Total	1.12	0.96	1.03	91

To summarise, we find that once the information on the experimental correlated systematics is included in the $\chi ^2$ definition, the differences at the PDF level are rather moderate and consistent with our baseline results, but that the numerical values of the $\chi ^2$ are higher. We also find that these $\chi ^2$ values are rather sensitive to the underlying correlation model, in particular to whether some specific sources of systematic errors are correlated or not between data bins. These poor $\chi ^2$ values deserve further investigation in the future in order to elucidate their underlying origin. It is in any case reassuring that the impact of the ATLAS direct photon data at the PDF level is mostly unaffected by this, validating the results presented in Sects. 4 and 5 of this work.

## B Reweighting study

An alternative strategy to quantify the impact of the ATLAS direct photon production measurements on the NNPDF3.1 global analysis is the Bayesian reweighting procedure [36, 37]. This technique allows one to determine the effects of a new dataset onto a Monte Carlo PDF set without refitting, and is therefore less computationally intensive. The only required inputs are the values of the figure of merit $\chi ^2_k$ to the new dataset computed using each of the $N_\mathrm{rep}$ replicas of the prior PDF set.

Given a dataset with $n_\mathrm{dat}$ data points, the Bayesian reweighting procedure assigns a weight $w_k$ to the *k*-th Monte Carlo replica given by

B.1 $$\begin{aligned} w_k = \frac{(\chi _k^2)^{\frac{1}{2}(n_\mathrm{dat}-1)}e^{-\frac{1}{2}\chi _k^2}}{\frac{1}{N_\mathrm{rep}}\sum _{k=1}^{N_\mathrm{rep}} (\chi _k^2)^{\frac{1}{2}(n_\mathrm{dat}-1)}e^{-\frac{1}{2}\chi _k^2}} ,\quad k=1,\ldots ,N_\mathrm{rep}\, , \end{aligned}$$

so that replicas that lead to theoretical predictions in disagreement with the new dataset (and that thus lead to larger $\chi ^2_k$) receive a small weight and are thus effectively discarded. The reweighting procedure also defines the effective number of replicas, $N_{\text {eff}}$, given by the Shannon entropy:

B.2 $$\begin{aligned} N_{\text {eff}} = \text {exp} \bigg \{\frac{1}{N_\mathrm{rep}}\sum _{k=1}^{ N_\mathrm{rep}} w_k \log (N_\mathrm{rep}/w_k) \bigg \} , \end{aligned}$$

which allow us to quantify how strongly the new data restricts the prior PDF set by how many replicas are left. The interpretation of Eq. (B.2) is that the smaller the ratio $N_\mathrm{eff}/N_\mathrm{rep}$, the higher the amount of new information that is being added by this specific experiment into the fit.

Using identical experimental data, kinematic cuts, and theoretical settings adopted to produce the NNPDF3.1 + ATLAS$\gamma$ set described in Sect. 4, we have reweighted the NNPDF3.1 NNLO set with $N_\mathrm{rep}=100$ replicas using Eq. (B.1). The resulting reweighted PDF set is denoted by NNPDF3.1 + ATLAS$\gamma$(rw). This reweighted set has been subsequently unweighted to a reduced number of replicas equal to the effective number of replicas $\widetilde{N}_\mathrm{rep}=N_\mathrm{eff}$, which can then be directly compared with the results of Sect. 4. For the total ATLAS direct photon production 8 TeV dataset, we find that $N_\text {eff} = 91$. In other words, $N_\text {eff}/N_\mathrm{rep}=0.91$, reflecting the moderate impact of the direct photon data on the NNPDF3.1 analysis

In Table 11 we provide the same comparison as in Table 3, now adding as well the $\chi ^2/N_\mathrm{dat}$ values corresponding to NNPDF3.1 + ATLAS$\gamma$(rw) set. We also indicate the value for $N_\mathrm{eff}$ corresponding to the total dataset. Note that we evaluate the reweighted $\chi ^2$ in each of the rapidity bin using the weights $\omega _k$ computed from the full dataset. The overall agreement between the fitted the reweighted versions of NNPDF3.1 + ATLAS$\gamma$(rw) is reasonably good, with residual differences traced back to the moderate loss of information involved in the reweighting. From Table 11 we observe that the value of $\chi ^2/N_\mathrm{dat}$ for the ATLAS direct photon data is 1.12 before the fit, reduced to 0.96 in the fit and 1.03 after reweighting, so both methods yield consistent results taking into account the statistical fluctuations of the $\chi ^2$ itself.

Concerning the comparison between fitting and reweighting at the PDF level, in Fig. 14 we show an updated version of Fig. 6 now adding also the results of the NNPDF3.1 + ATLAS$\gamma$(rw) set. We see that the results of both gluons are close to each other, and in particular that both the fitted and reweighted versions of NNPDF3.1 + ATLAS$\gamma$(rw) exhibit the clear preference for a somewhat softer gluon at large *x*. In terms of the gluon PDF uncertainty, also here the results of the fitted and reweighted sets are reasonably similar. Note that in particular in the large-*x* region, , the reduction of the PDF uncertainties as compared to the baseline determined using the two methods is identical.

To summarize, the fitted and the reweighted versions of the NNPDF3.1 + ATLAS$\gamma$ PDF set are in good agreement with each other. This agreement could be further improved if a larger prior would have been used, specifically the NNPDF3.1 NNLO set with $N_\mathrm{eff}=1000$ replicas. This is however not required here, since our goal was limited to providing a validation of the qualitative features of the fit results by means of an independent technique.

## Footnotes

1 These fast NLO APPLgrids, matching the selection cuts and the binning of the ATLAS 8 and 13 TeV direct photon production measurements, are available from the authors.
